# ABO Antibody Titer Dynamics and Optimization in Incompatible Renal Transplants Using Therapeutic Plasma Exchange

**DOI:** 10.7759/cureus.91015

**Published:** 2025-08-26

**Authors:** Anubha Srivastava, Priti Elhence, Bharat Singh, Anupam Verma, Prashant Agarwal, Dheeraj Khetan, Rajendra K Chaudhary, Aneesh Srivastava, Narayan Prasad

**Affiliations:** 1 Transfusion Medicine, Dr. Ram Manohar Lohia Institute of Medical Sciences, Lucknow, IND; 2 Transfusion Medicine, Sanjay Gandhi Postgraduate Institute of Medical Sciences, Lucknow, IND; 3 Urology, Sanjay Gandhi Postgraduate Institute of Medical Sciences, Lucknow, IND; 4 Nephrology, Sanjay Gandhi Postgraduate Institute of Medical Sciences, Lucknow, IND

**Keywords:** abo-incompatible kidney transplant, antibody-mediated rejection, immunohematology, patient outcomes, therapeutic plasma exchange, transfusion medicine

## Abstract

Background

ABO-incompatible kidney transplantation (ABOi KT) expands access to living-donor organs but requires careful control of pre-existing anti-ABO isoagglutinins to minimize antibody-mediated rejection (ABMR). The present study was designed with the following objectives:

The primary objective was to assess the relationship between baseline anti-ABO IgG titers (≥ 64 vs. < 64) and the intensity of therapeutic plasma exchange (TPE) required pre- and post-transplant, as well as ABMR incidence. The secondary objective was to evaluate graft function, patient and graft survival, and the frequency and severity of TPE-related adverse events using a standardized TPE-based desensitization protocol in a resource-limited center.

Methods

In this prospective single‑center study, 20 dialysis‑dependent recipients of live‑related ABOi KT received tacrolimus‑based immunosuppression, TPE, intravenous immunoglobulin, rituximab, and anti‑thymocyte globulin. ABO IgG and immunoglobulin M titers were monitored serially. Patients were stratified by high (≥64) versus low (<64) baseline IgG titers. Primary outcomes were pre‑ and post‑transplant TPE requirements and ABMR incidence; secondary outcomes included graft function, patient and graft survival, and TPE‑related adverse events.

Results

Across the cohort, 144 TPE sessions were performed. Recipients with high baseline IgG titers required more pretransplant TPE than those with low titers (mean 5.2 ± 1.75 standard deviation vs 3.4 ± 1.3; p = 0.02), whereas post‑transplant TPE use was comparable. ABMR occurred in 45 % of patients and was more frequent in the high‑titer group, though the difference was not statistically significant. Most recipients maintained stable graft function at 24 months, patient survival was high, and TPE‑related adverse events were infrequent and mild.

Conclusions

Higher baseline IgG titers increase the need for pretransplant desensitization but do not drive additional post‑transplant TPE. An individualized, TPE‑centered protocol can safely and effectively enable ABOi KT in resource‑constrained settings, underscoring the value of risk‑adapted strategies and real‑time immunomonitoring to optimize transplant outcomes.

## Introduction

ABO‑incompatible kidney transplantation (ABOi KT) expands the living‑donor pool by permitting grafts across the blood‑group barrier; however, pre‑formed recipient anti‑A and anti‑B antibodies can trigger antibody‑mediated rejection (ABMR). Successful engraftment, therefore, relies on intensive pre‑transplant desensitization and sustained post‑transplant immunosuppression. With modern protocols, ABOi KT can raise living‑donor transplant rates by as much as 30 % and shorten wait times, outcomes once unattainable when ABO incompatibility was considered prohibitive [1].

Direct antibody removal is central to both perioperative phases. Therapeutic plasma exchange (TPE), double‑filtration plasmapheresis, and immunoadsorption each eliminate circulating isoagglutinins; TPE, which replaces separated plasma with colloid and/or crystalloid solutions, is the most widely used. Combined with potent immunosuppression, TPE now yields graft and patient survival comparable to ABO‑compatible (ABOc) transplantation [2]. The American Society for Apheresis (ASFA) classifies TPE as a Category I indication for desensitization in ABOi KT and Category II for ABMR treatment [3]. Because baseline antibody burden varies, many recipients need multiple sessions, adding procedural complexity and risk.

Perioperative monitoring of immunoglobulin G (IgG) and immunoglobulin M (IgM) anti‑A/B titers guides treatment intensity. Column agglutination technology (CAT) offers faster, more objective measurement than the conventional tube technique. High baseline IgG isoagglutinin levels may predict stronger post‑transplant immune responses and greater ABMR risk [4], but evidence remains limited, particularly from resource‑constrained settings.

Successful engraftment in ABOi KT depends on effective pre-transplant desensitization and vigilant post-transplant monitoring to prevent ABMR. While high baseline anti-ABO IgG titers have been suggested as predictors of increased desensitization requirements and higher ABMR risk, evidence from resource-limited settings remains limited. Additionally, there is a need to evaluate the clinical performance and safety of standardized therapeutic plasma exchange (TPE)-based protocols in such environments.

Therefore, the present study was designed with the following objectives: (i) Primary objective: Assess the relationship between baseline anti-ABO IgG titers (≥ 64 vs. < 64) and the intensity of TPE required pre- and post-transplant, as well as the incidence of ABMR; (ii) Secondary objective: Evaluate graft function, patient and graft survival, and the frequency and severity of TPE-related adverse events using a standardized TPE-based desensitization protocol in a resource-limited center.

## Materials and methods

This was a prospective observational study conducted at the Department of Transfusion Medicine, Sanjay Gandhi Postgraduate Institute of Medical Sciences, Lucknow, Uttar Pradesh, India, from May 2015 to February 2018. The study was approved by the Institutional Ethics Committee of the Sanjay Gandhi Postgraduate Institute of Medical Sciences, Lucknow (2015-21-MD-EXP, dated May 6, 2015).

Eligibility criteria

Inclusion criteria included adult patients (≥18 years) with end-stage renal disease (ESRD) on dialysis who underwent live-related ABOi KT between 2015 and 2018 and consented to the standardized desensitization protocol. Exclusion criteria included pediatric patients (<18 years), patients undergoing ABO-compatible kidney transplantation, and patients who did not complete the desensitization protocol or had incomplete follow-up data.

Preconditioning and immunosuppression protocol

**Figure 1:**
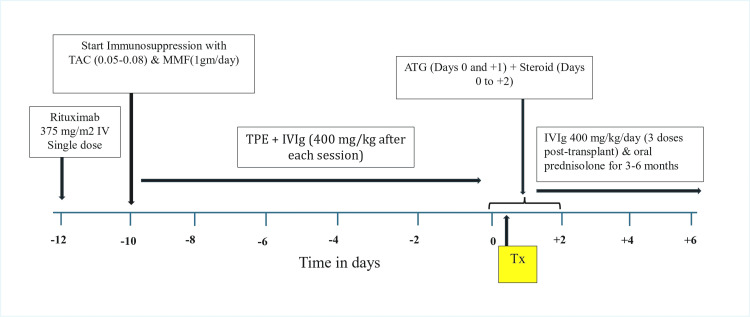
Preconditioning protocol for ABO-incompatible kidney transplantation. IVIg, intravenous immunoglobulin; ATG, anti-thymocyte globulin; TAC, tacrolimus; MMF, mycophenolate mofetil;  TPE+IVIg, plasma exchange followed by immunoglobulin; Tx, transplant

All included adult recipients received a uniform desensitization and immunosuppression protocol. For preconditioning, rituximab (375 mg/m² IV) was administered on Day -12. Tacrolimus (0.05-0.08 mg/kg twice daily) and mycophenolate mofetil (1000 mg daily) were started on Day -10. TPE with IVIg (400 mg/kg after each session) was initiated concurrently to reduce anti-ABO isoagglutinins. TPE frequency was individualized to achieve a pretransplant anti-ABO IgG titer ≤ 8. Induction included anti-thymocyte globulin (1.5 mg/kg IV on Days 0 and +1) and methylprednisolone pulses (500 mg, 250 mg, and 125 mg IV on Days 0 to +2), followed by oral prednisolone immediately post-transplant (starting at 20 mg/day and tapered over three to six months). Three more doses of IVIg were administered post-transplant (400 mg/kg/day on Days +1 to +3) while TPE was repeated post-transplant only in cases of titer rebound or ABMR. Tacrolimus and MMF were continued with dose adjustments based on therapeutic levels and tolerability. Tacrolimus trough levels were maintained at 8-12 ng/mL during the first three months of the post-transplant period, then reduced to 5-8 ng/mL. MMF doses were adjusted for tolerability. Rituximab and ATG were not repeated during follow-up. Figure 1 shows the preconditioning protocol.

Therapeutic plasma exchange

TPE was initiated on Day -10 prior to transplantation, concurrently with the start of immunosuppressive therapy. The indication for pre-transplant TPE was desensitization, aiming to reduce anti-ABO IgG and IgM isoagglutinin titers to ≤ 8. Sessions were performed every one to two days based on titer response, and TPE was discontinued once the target titer of ≤ 8 was achieved, immediately before transplant. The number of sessions varied depending on baseline titer levels and individual response to therapy.

Post-transplant TPE was performed in patients with antibody rebound (IgG ≥ 8) or clinically suspected/biopsy-proven ABMR. The procedure was continued until resolution of the rejection episode, defined by: return of titers to ≤ 8, improvement or stabilization of serum creatinine, and absence of ongoing clinical or histological evidence of rejection.

All TPE sessions were conducted using the membrane filtration method. Replacement fluids included fresh frozen plasma (FFP) and albumin, with anticoagulation provided using low-molecular-weight heparin. The number of pre- and post-transplant TPE procedures per patient was recorded, along with any associated adverse events. All patients undergoing TPE received intravenous calcium gluconate before and during the procedure to prevent citrate-induced hypocalcemia, with 79.2% receiving calcium alone. Additional premedications were used selectively based on prior adverse reactions: hydrocortisone (100 mg IV) in 3.5% of procedures for patients with a history of hypersensitivity to plasma products, and pheniramine maleate (45.5 mg IV) in 17.5% of procedures for those who had experienced pruritus, urticaria, or other mild allergic symptoms during plasma-containing replacement therapy.

The efficacy of TPE was assessed by monitoring changes in anti-A and anti-B antibody levels in conjunction with immunosuppressive therapy. We also recorded adverse events related to TPE procedures.

Immunohematological workup

ABO and Rh(D) blood grouping of both recipient and donor was performed using column agglutination technology (CAT) (DiaMed Holding AG, Cressier, Switzerland). Donor-recipient ABO incompatibilities were defined as major incompatibility (blood group B, A, or AB donor to O recipient; AB donor to A or B recipient) or bidirectional incompatibility (blood group A donor to B recipient, or vice versa). Peripheral B-cell (CD19⁺) enumeration was not routinely performed following Rituximab administration due to logistical limitations.

IgM and IgG ABO Antibody Titration

IgM and IgG anti-A/B isoagglutinin titers were measured separately using CAT (DiaMed Holding AG). Serum or plasma samples were serially diluted in two-fold increments and tested against pooled 1% suspensions of A and B reagent red cells prepared in a low-ionic-strength solution (LISS). IgM titers were determined using sodium chloride (NaCl) gel cards, which detect naturally occurring cold-reactive antibodies through direct agglutination. IgG titers were measured using antihuman globulin (AHG) gel cards, enabling selective detection of IgG-class antibodies.

The titer was defined as the highest dilution showing visible agglutination (≥1+ reaction). at baseline, before and after each TPE procedure, daily for the first two weeks post-transplant, weekly for the following month, and then at two, three, six, and 12 months post-transplant, as well as whenever clinically indicated, in order to promptly detect antibody rebound and guide further treatment.

At the time of transplant, ABO antibody titers were required to be < 8. To assess the impact of baseline titers on TPE requirements, patients were divided into two groups based on their starting IgG titers: ≥ 64 and < 64.

Outcome definitions and follow-up

Immediate ABMR

Immediate ABMR was defined as biopsy‑proven or clinically suspected rejection occurring within the first seven days after transplantation. Kidney allograft biopsy was performed only when clinically indicated, not as a routine protocol. Indications included an unexplained ≥ 25% rise in serum creatinine from baseline, anti-ABO antibody rebound (IgG ≥ 8) with graft dysfunction, or clinical signs suggestive of acute rejection such as reduced urine output or graft tenderness. Diagnostic criteria included a sudden rise in serum creatinine, detection of donor‑specific antibodies or anti‑ABO antibody rebound, and Banff‑classified histopathological changes consistent with ABMR [5].

Partial Recovery and Graft Loss

Partial recovery of graft function was defined as a sustained post‑treatment decrease in serum creatinine of at least 30 % from the peak rejection value, without a return to the patient’s baseline level, and persisting for a minimum of three months [6]. Graft loss refers to irreversible allograft failure necessitating a permanent return to dialysis or re‑transplantation [7].

Follow‑Up

All recipients were followed for 24 months with scheduled clinic visits that included a physical examination, serum creatinine measurement, and repeat anti‑A/B antibody titration. Additional visits were arranged if clinical or immunological concerns arose.

Statistical analysis

Analyses were conducted using SPSS Statistics for Windows, version 18.0 (SPSS Inc., Chicago, Illinois, United States). Independent-samples t-tests were used to compare the number of TPE procedures between high and low baseline IgG groups, while Fisher’s exact test evaluated the association between baseline IgG category and ABMR incidence. Continuous variables are reported as mean ± standard deviation (SD), and a two-sided p-value <0.05 was considered statistically significant.

## Results

Patient demographics and characteristics

During the study period, 20 patients underwent live-related ABOi KT. All patients were dialysis-dependent at the time of admission. Demographic and clinical characteristics of the recipients are presented in Table 1. All transplant recipients were men. The ages of the related kidney donors ranged from 22 to 66 years (median: 44 years), and 15% of the donors were men. The most common underlying cause of end-stage renal disease was rapidly progressive renal failure or glomerulonephritis.

**Table 1:** Patient demographics and characteristics (N=20) RPRF, rapid progressive renal failure; RPGN, rapid progressive glomerulonephritis; ADPKD, autosomal dominant polycystic kidney disease; IgG, immunoglobulin G; IgM, immunoglobulin M; Pre-KT, pre-kidney transplantation; M, male; F, female; SD, standard deviation; HLA, human leukocyte antigen.

Parameter		Kidney Recipients (N=20)
Age (years), range (mean ± SD)		7 to 75 (37 ± 15.2)
Gender (M:F), n (%)		20:0 (100%:0%)
Pre-transplant dialysis period (months), range (mean ± SD)		7–60 (17.5 ± 11.83)
Hospital stay during transplant (months), range (mean ± SD)		1–3 (1.38 ± 0.58)
Native Kidney Disease	RPRF/RPGN	12
	Chronic Interstitial Nephropathy	2
	Diabetic Kidney Disease	3
	ADPKD	2
	IgA Nephropathy	1
Post-transplant follow-up (months), mean ± SD		38.1 ± 17.07
Blood Group, n (%)	O Rh D Positive	7 (35)
	A Rh D Positive	8 (40)
	B Rh D Positive	5 (25)
ABO incompatibility, n	A to B	3
	B to A	6
	B to O	4
	A to O	2
	AB to O	1
	AB to A	2
	AB to B	2
Initial Baseline IgM titer, median (range)		16 (1–128)
Initial Baseline IgG titer. median (range)		48 (2–256)
Pre-KT IgM titer, median (range)		1 (0–8)
Pre-KT IgG titer. median (range)		4 (1–16)
HLA Mismatch	1 allele	1
	2 alleles	2
	3 alleles	3
	4 alleles	3
	5 alleles	7
	6 alleles	4

Changes in IgG and IgM isoagglutinin titers

**Figure 2:**
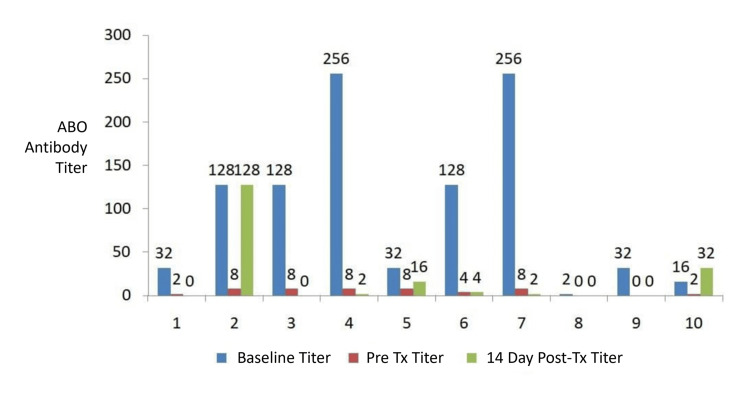
Baseline, pre-transplant, and 14-day post-transplant IgG ABO antibody titers in patients 1–10 undergoing ABO-incompatible kidney transplantation. IgG, immunoglobulin G; Tx, transplant.

**Figure 3:**
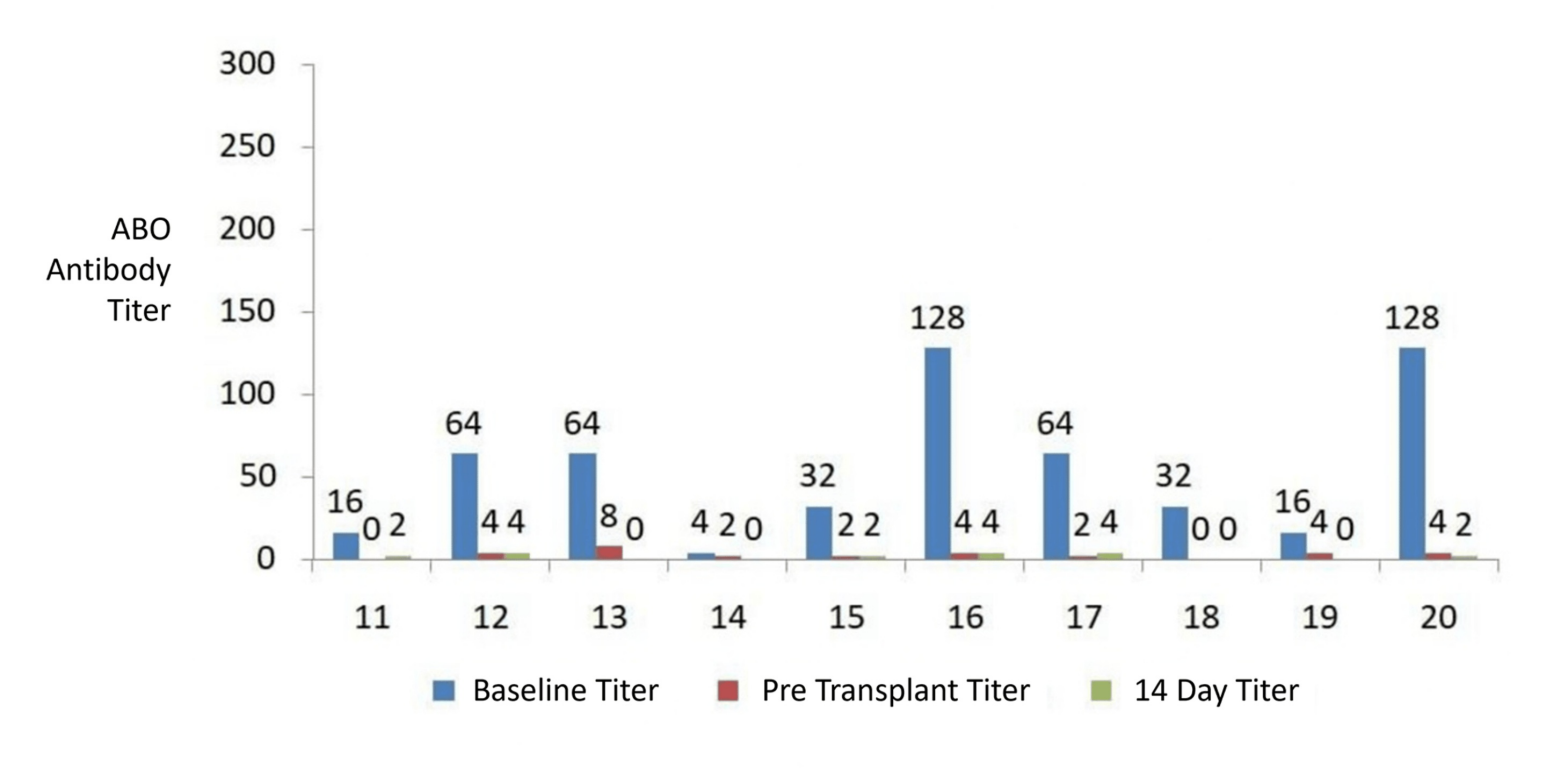
Baseline, pre-transplant, and 14-day post-transplant IgG ABO antibody titers in patients 11–20 undergoing ABO-incompatible kidney transplantation. IgG, immunoglobulin G

A total of 768 ABO IgG and IgM titration tests were performed for the 20 patients during the pretransplant and post-transplant follow-up periods. Figures 2, 3 show the relevant ABO antibody titers at baseline, immediately before transplantation, and 14 days post transplant. Baseline IgM titers ranged from 1 to 128, and baseline IgG titers ranged from 2 to 256. Ten patients (50%) had high initial IgG titers, defined as ≥ 64 at the time of admission. All patients achieved pretransplant IgG and IgM titers ≤ 8. No immediate post-transplant increase in antibody titers was observed in 17 patients (85%).

Therapeutic plasma exchange

Of the 20 patients, 18 required pretransplant TPE. A total of 86 pre-transplant TPE procedures were performed (mean ± SD: 4.3 ± 2.36; range: 0-10). Post-transplant TPE was required in 14 patients whose titers increased to ≥1:8, with 58 procedures conducted in total (mean ± SD: 2.9 ± 2.15; range: 0-5). Across all patients, a total of 144 TPE procedures were performed (mean ± SD: 7.2 ± 3.69; range: 0-12), including 86 pretransplant and 58 post-transplant procedures, as shown in Table 2.

**Table 2:** Details of baseline titer, TPE procedures performed, and patient outcomes ABMR, antibody-mediated rejection; TPE, therapeutic plasma exchange; HLA, human leukocyte antigen; SD, standard deviation; Txp, transplant

Patient	HLA Cross Match(Pos/Neg)	HLA Mismatch	Baseline ABO titer (High ≥64, Low <64)	Antibody-Mediated Rejection	TPE procedures (N=144) (Mean ± SD = 7.2 ± 3.69, Range: 0-12)		Outcome
					Pre-Txp (N=86) (Mean ± SD = 4.3 ± 2.36, Range: 0-10)	Post-Txp (N=58) (Mean ± SD = 2.9 ± 2.15, Range: 0-5)	
1	Positive	3/6	128 (High)	ABMR 1 month after transplant	5	5	Recovery
2	Positive	5/6	32(Low)	No ABMR	5	0	Recovery
3	Negative	5/6	16 (Low)	Immediate ABMR	4	4	Recovery
4	Negative	2/6	128 (High)	Immediate ABMR	7	5	Partial Recovery
5	Negative	1/6	64 (High)	Immediate ABMR	6	4	Partial Recovery
6	Negative	6/6	32 (Low)	No ABMR	6	4	Recovery
7	Negative	5/6	16 (Low)	No ABMR	4	0	Recovery
8	Negative	5/6	128 (High)	Immediate ABMR & 2-month post-Txp ABMR	6	5	Recovery
9	Negative	2/6	256 (High)	High titer, 4 months ABMR	4	4	Recovey
10	Negative	5/6	4 (Low)	No ABMR	0	0	Recovery
11	Negative	6/6	64 (High)	No ABMR	3	2	Recovery
12	Negative	3/6	2 (Low )	No ABMR	0	0	Recovery
13	Negative	6/6	256 (High)	Immediate ABMR	10	2	Recovery
14	Negative	4/6	32 (Low)	Low titer, 5 months ABMR	2	4	Recovery
15	Negative	5/6	64 (High)	Immediate ABMR	3	0	Recovery
16	Negative	6/6	32 (Low)	Immediate ABMR	6	6	Recovery
17	Negative	6/6	128 (High)	No ABMR	5	4	Recovery
18	Negative	4/6	128 (High)	Immediate ABMR	3	4	Recovery
19	Negative	4/6	32 (Low)	Immediate ABMR	2	5	Partial Recovery
20	Negative	3/6	16 (Low)	No ABMR	5	0	Recovery

Patients with high baseline IgG titers required a mean of 5.2 ± 1.75 pretransplant TPE procedures, while those with lower baseline titers required a mean of 3.4 ± 1.3 procedures (p = 0.02). The number of post-transplant TPE procedures was 3.1 ± 1.8 in the high-titer group and 2.3 ± 2.5 in the low-titer group (p = 0.5), as shown in Table 3.

**Table 3:** Comparison of TPE procedures based on baseline IgG titers *Unpaired t-test, p-value ≤ 0.05 is considered significant. TPE, therapeutic plasma exchange; IgG, immunoglobulin G.

Parameter	High IgG Titer Group (Mean ± SD)	Low IgG Titer Group (Mean ± SD)	P-Value*	Statistical Significance
Pre-transplant TPE procedures	5.2 ± 1.75	3.4 ± 1.3	0.02	Significant
Post-transplant TPE procedures	3.1 ± 1.8	2.3 ± 2.5	0.5	Not Significant

The mean plasma volume exchanged per TPE procedure was 2.5 liters. FFP and albumin were both used as replacement fluids. The average duration for a two-volume exchange was approximately two to two-and-a-half hours. Premedications included calcium alone in 114 procedures (79.2%), calcium and hydrocortisone in five procedures (3.5%), and calcium with pheniramine maleate (Avil) in 25 procedures (17.5%). Vascular access was obtained via the internal jugular vein in two patients (10%) and via an arteriovenous fistula in 18 patients (90%). LMWH was used as the anticoagulant in all procedures. Adverse effects during TPE procedures were minimal. The most commonly reported symptoms were nausea (22 procedures, 15.2%), followed by hypotension and itching (17 procedures, 11.8%), pain (12 procedures, 8.3%), and hypertension (five procedures, 3.4%).

Association between baseline IgG titers and ABMR

ABMR occurred in eight of 10 patients with high baseline IgG titers and in four of 10 patients with low titers (Table 4). Although the incidence was higher in the high‑titer group (80 % vs 40 %), the difference did not reach statistical significance (Fisher’s exact test, p = 0.170). 

**Table 4:** Association between baseline IgG titers and ABMR *Two-tailed test, p-value ≤ 0.05 is considered significant. IgG, immunoglobulin G; ABMR, antibody-mediated rejection

IgG Level	ABMR	No ABMR	Total	P-Value
High IgG	8	2	10	0.170*
Low IgG	4	6	10	
Total	12	8	20	

Of the 20 recipients, nine (45 %) experienced acute ABMR. Within this group, one episode occurred two months after transplantation, and three additional episodes were documented at one, four, and five months, respectively. All episodes were managed with intensive post-transplant TPE. Of these 12 patients, nine achieved full recovery of renal function, while three exhibited partial recovery. One of the patients with partial recovery died from a coronary event. The remaining 17 patients maintained stable renal function at the two-year follow-up, as shown in Table 2.

At one month post-transplant, the mean serum creatinine level was 1.7 ± 2.0 mg/dL, and most patients demonstrated satisfactory early graft function.

At median follow-up of 24 months, patient survival was 95% (95% CI, 85.4-100), with a single death due to a coronary event. Graft survival at this time was 85% (95% CI, 69.4-100), reflecting three cases of graft dysfunction (one in the deceased patient and two with partial recovery). The mean serum creatinine at final follow-up was 2.1 ± 1.9 mg/dL.

## Discussion

This study highlights the critical role of TPE in the desensitization and post-transplant management of patients undergoing ABOi KT. A key finding was the significantly greater requirement for pretransplant TPE in patients with high baseline IgG titers (≥64), who underwent a mean of 5.2 ± 1.75 procedures, compared to 3.4 ± 1.3 in those with lower titers (p = 0.02). This aligns with the findings of Won et al., who reported that higher isoagglutinin titers and slower reduction rates were associated with an increased risk of titer rebound post-transplantation, necessitating more intensive preconditioning strategies [8]. Similarly, Naciri Bennani et al. found that patients with high baseline titers (≥ 256) required more pretransplant apheresis sessions (10.5 ± 3.5) than those with lower titers [9].

Interestingly, no statistically significant difference was observed in the number of post-transplant TPE sessions between the high- and low-titer groups (3.1 ± 1.8 vs. 2.3 ± 2.5; p = 0.5), suggesting that effective pre-transplant desensitization may reduce the need for postoperative interventions. 

A higher incidence of ABMR was observed in recipients with high baseline IgG titers (80 %) than in those with low titers (40%); however, the difference was not statistically significant (p = 0.170), likely due to the limited sample size. Earlier studies have likewise linked elevated isoagglutinin levels to greater ABMR risk in ABOi KT [8], implying that stronger humoral responses call for more intensive desensitization. Although our result lacks statistical significance, it remains clinically meaningful, reinforcing the importance of thorough pre‑transplant immunologic assessment and of larger studies to confirm the prognostic value of baseline IgG titers and to refine risk‑adapted desensitization protocols.

Despite successful desensitization, approximately 15% of patients in this study experienced an immediate post-transplant rise in antibody titers. This highlights the ongoing risk of titer rebound and underscores the importance of precise monitoring and timely postoperative intervention. These results support the growing emphasis on individualized desensitization protocols, as outlined in the ASFA 2023 guidelines [3]. Future studies incorporating real-time immunomonitoring and extended follow-up could help define optimal TPE timing and endpoints, improving outcomes and resource utilization.

In this cohort, the frequency of pretransplant TPE (86 procedures; mean ± SD: 4.3 ± 2.36) exceeded that of post-transplant procedures (58 procedures; mean ± SD: 2.9 ± 2.15). This is consistent with widely adopted practices emphasizing aggressive pretransplant desensitization to lower anti-ABO antibody titers to ≤ 8 or ≤ 4, depending on institutional protocols [10,11]. The observed range of TPE sessions (0-10 pre-transplant and 0-5 post-transplant) reflects the individualized nature of desensitization, which is often tailored to baseline isoagglutinin titers and clinical response.

Patients with slower antibody clearance or higher baseline titers may require more procedures [4]. In this study, several patients required post-transplant TPE due to antibody rebound or early ABMR, which remains a known risk despite adequate preconditioning [12]. Close postoperative monitoring of anti-A/B titers is essential, as titer rebound has been linked to increased ABMR incidence and poor graft outcomes [13]. Our study’s mean of 2.9 ± 2.15 post-transplant TPE procedures reflects the need for continued vigilance in the immediate postoperative period.

The cumulative TPE burden (mean: 7.2 ± 3.69 per patient) aligns with international experiences, particularly in Asian transplant centers, where living-donor ABOi KT is increasingly performed [14]. In our cohort, the overall incidence of ABMR was 45% in the immediate postoperative period, with additional cases noted at one, two, four, and five months. Despite this, 85% of patients achieved full or partial recovery. These findings reaffirm the role of TPE in the management of acute ABMR. Although the ABMR rate in this study is higher than the 11% reported by Lipshutz et al. [15], it is comparable to findings by Uchida et al. [16] and Setoguchi et al. [17], who reported ABMR rates of 32% and 27%, respectively, in ABOi cohorts. Variability in desensitization protocols, patient selection, and baseline titers may account for these discrepancies.

Two years after transplantation, patient survival was 95 % and graft survival was 85 %, indicating favorable short‑ to mid‑term outcomes with the structured desensitization protocol used for ABOi KT. Occasional graft dysfunction nonetheless persists, emphasizing the need for ongoing vigilance beyond the early postoperative period. These results are consistent with previous reports demonstrating improved graft survival and reduced ABMR incidence when early and aggressive immunomodulation, including TPE, is employed [18,19]. The three patients with only partial recovery may have experienced subclinical or overt ABMR, possibly compounded by comorbid vascular risk factors or delayed rescue therapy. 

TPE was well tolerated in this cohort. Adverse effects were generally mild, with nausea (15.2%) and hypotension (11.8%) being most common. These rates are comparable to those reported by Shemin et al. and Norda et al., who noted mild complications in 7.7% to 7.4% and hypotension in less than 1% of procedures, respectively [20,21]. Despite the high number of TPE sessions (n = 144), no severe procedure-related complications were observed. This supports safely incorporating TPE into ABOi KT protocols, even in resource-limited settings.

Limitations

This study has several limitations. The small, single-center, retrospective design restricts external validity and leaves the findings susceptible to unmeasured confounders. The absence of an ABO-compatible control cohort and clinician-dependent thresholds for initiating TPE or diagnosing ABMR may introduce selection and observer bias. Antibody titers were assessed with a single-column agglutination platform without external quality control, which may limit comparability with other laboratories. All recipients were men from one region of India, further constraining generalizability. Follow-up of only two years precludes evaluation of late graft loss, rare severe adverse events, cost-effectiveness, and long-term quality-of-life outcomes. One of the limitations of this study was the absence of serial peripheral CD19⁺ B-cell monitoring after rituximab administration. While effective desensitization and clinical outcomes suggested adequate B-cell depletion, future studies could incorporate real-time CD19⁺ enumeration to better individualize B-cell-targeted therapies and monitor reconstitution dynamics post-transplant. Another limitation of this study is the absence of serial estimated glomerular filtration rate (eGFR) measurements, which precluded detailed charting of early post-transplant renal function; only serum creatinine values were recorded. 

## Conclusions

This study evaluated the effectiveness and safety of TPE in managing ABOi KT, focusing on the relationship between baseline antibody titers and the intensity of desensitization required. The findings demonstrate that higher pretransplant isoagglutinin titers are associated with a greater need for TPE before transplantation but not necessarily with increased post-transplant interventions. The findings also highlight the clinical relevance of baseline IgG titers as potential predictors of ABMR risk, warranting individualized protocols and vigilant post-transplant monitoring. Most patients experienced successful graft function, and adverse effects were minimal, supporting the role of TPE as a cornerstone of desensitization and post-transplant care in ABO-incompatible recipients.

These results support broader adoption of individualized desensitization protocols, particularly in settings where living donor options are available but blood group incompatibility poses a barrier. The study affirms the feasibility of ABOi KT in resource-constrained environments and highlights the critical role of transfusion medicine teams. Future research should explore real-time immunomonitoring and standardized titer measurement to refine TPE timing further, reduce complications, and optimize long-term outcomes.
